# Efficacy and Safety of Acupuncture in the Treatment of Poststroke Insomnia: A Systematic Review and Meta-Analysis of Twenty-Six Randomized Controlled Trials

**DOI:** 10.1155/2022/5188311

**Published:** 2022-03-19

**Authors:** Liang Zhou, Xiuwu Hu, Zhen Yu, Lihui Yang, Renhong Wan, Haolin Liu, Ying Wang

**Affiliations:** ^1^Nanchang Hongdu Hospital of Traditional Chinese Medicine, Nanchang 330000, Jiangxi, China; ^2^Department of Encephalopathy, Tianjin Academy of Traditional Chinese Medicine Affiliated Hospital, Tianjin 300120, China; ^3^Tianjin University of Traditional Chinese Medicine, Tianjin 301617, China

## Abstract

**Objective:**

To evaluate the efficacy and safety of acupuncture in the treatment of poststroke insomnia.

**Methods:**

PubMed, the Cochrane Library, Embase, Web of Science, China Biology Medicine (CBM), CNKI, VIP, and Wanfang databases were searched by computer from their inception to April 29, 2021, for collecting all randomized controlled trials of acupuncture in the treatment of poststroke insomnia. After two reviewers independently screened the literature, extracted the data, and evaluated the risk of bias in the included studies, the data were analyzed by RevMan 5.3 and STATA 16.0. The quality of outcomes was evaluated by the Grading of Recommendations Assessment, Development and Evaluation (GRADE).

**Results:**

A total of 26 studies with 1874 cases were included, which had 942 cases in the treatment group and 932 cases in the control group. Meta-analysis results showed that, compared with oral medications alone, acupuncture alone or acupuncture combined with oral medications could improve the clinical effective rate and the sleep quality of patients, and the combined effects were RR = 1.21; 95% CI: 1.15, 1.27; *P* < 0.00001 and MD = 3.41; 95% CI: 2.40, 4.41; *P* < 0.00001, respectively. As for adverse reactions, the incidence of acupuncture alone or acupuncture combined with oral drugs was lower than that of oral drugs alone, which was safer and the combined effect was RR = 0.21; 95% CI: 0.09, 0.48; *P*=0.0002. Sensitivity analysis showed that the results were stable. We evaluated the quality of evidence with the GRADE system; the clinical effective rate was rated as “LOW,” the evidence grade of PSQI score was “LOW,” and the evidence grade of adverse reactions was “Very LOW.”

**Conclusion:**

Acupuncture alone or acupuncture combined with oral drugs is more effective and safer than oral drugs alone in the treatment of poststroke insomnia, which is suitable to promote in clinical practice.

## 1. Introduction

Stroke, a common cerebrovascular disease, is the second leading cause of death in the world [1]. Stroke patients often suffer a series of symptoms, including limb dysfunction, aphasia, dysphagia, cognitive impairment, and insomnia [2–4]. Poststroke insomnia, a sleep disorder that occurs in patients with stroke during the recovery and sequelae periods, is mainly characterized by difficulty falling asleep, early awakening, or short sleep time. Studies have found that [5, 6] the incidence of insomnia in stroke patients is 34%–67%. Long-term insomnia will not only cause a decline in the stroke patients' quality of life but also affect their physical and mental health as well as the recovery of limb function and, at the same time, increase the risk of coronary heart disease, diabetes, and hypertension to a certain extent, inducing secondary stroke, which brings burden to the family and society [7, 8].

The pathogenesis of poststroke insomnia is not yet clear. Oral benzodiazepines are often used in clinical practice. Although these drugs work quickly, there is obvious tolerance as well as adverse reactions and even significant toxicity and side effects, which limit the long-term use of these drugs [9, 10]. Therefore, seeking safe and effective treatment methods is crucial to the sleep health of stroke patients. The existing clinical trials [11] have shown that acupuncture has more advantages than western medicine, with rapid effects, safety, and convenience and no obvious adverse reactions, which is more and more favored by insomnia patients and has become one of the effective ways to treat insomnia. At present, there are many clinical trials of acupuncture in the treatment of poststroke insomnia, but the interventions adopted have many forms and the quality of literature is uneven, which hinders the clinical promotion of acupuncture in patients with poststroke insomnia to a certain degree. Hence, our study collected the clinical trials of acupuncture in the treatment of poststroke insomnia for meta-analysis and compared the efficacy and safety of acupuncture against poststroke insomnia to provide a reliable evidence-based basis for the clinical application of acupuncture in patients with poststroke insomnia.

## 2. Methods

### 2.1. Protocol Registration

According to the guidance of the Preferred Reporting Items for Systematic Review and Meta-Analysis Protocols (PRISMA-P) [12] (see Table S1 for the PRISMA checklist), we have taken this protocol of systematic review and meta-analysis to be drafted. The protocol and registration information are available at https://www.crd.york.ac.uk/prospero/display_record.php?ID=CRD42021269736 (registration number: CRD42021269736).

### 2.2. Ethics

Because this study is an analysis of the literature, which does not need to recruit patients nor does it involve patient privacy, the informed consent of the patient and the approval of the ethics committee are not required.

### 2.3. Eligibility Criteria

#### 2.3.1. Study Type

All randomized controlled trials (RCTs) of acupuncture in the treatment of poststroke insomnia were eligible, regardless of blinding, publication status, or region, but the language was restricted to Chinese and English.

#### 2.3.2. Types of Participants

All patients had clear diagnostic criteria for stroke and insomnia, without serious abnormalities of heart, liver, and kidney function, and the nationality, age, gender, and condition of the patient were unlimited.

#### 2.3.3. Interventions

The control group was treated with oral sleeping western medicine (unlimited kind and dose). The experimental group adopted acupuncture with the drug of the control group or acupuncture related therapy alone.

#### 2.3.4. Outcome Indicators

*(1) Primary Outcomes*. For clinical effective rate, the clinical efficacy was evaluated according to *Guiding Principles for Clinical Research of New Chinese Medicines* [13]. For recovery, the patient's sleep returned to normal at night or the sleep duration was ≥6 hours, and the sleep was deep and dreamless, and the patient was energetic after waking up; for significant effect, the patient's nocturnal sleep was significantly improved or the sleep duration increased ≥3 hours, and the sleep depth increased significantly; for effectiveness, nocturnal sleep time improved or the sleep duration increased ≥3 hours; for ineffective, the patient's sleep at night did not improve or even got worse. Clinical effective rate = (recovery number + significant effect number + effectiveness number)/total number. *(2) Secondary Outcomes*. The first is Pittsburgh sleep quality index (PSQI) and the second is adverse reactions. Meeting any of the above indicators could be included in the analysis.

### 2.4. Exclusion Criteria

The exclusion criteria were as follows: (1) For repetitive publications, only the ones with the most complete data and the highest quality were included. (2) Articles with incomplete data that still could not be obtained after contacting the authors were excluded. (3) Studies with obvious data errors or no relevant outcome indicators were excluded. (4) The intervention group was acupuncture combined with other treatment methods, such as traditional Chinese medicine formulae and rehabilitation training. (5) Studies without clear disease diagnostic criteria in the articles were excluded. (6) Studies with the randomization method rated as high risk were excluded [14].

### 2.5. Search Strategy

The retrieval method employed the combination of Medical Subject Headings (MeSH) terms and free terms, involving acupuncture, acupuncture and moxibustion, electroacupuncture, apoplexy, stroke, cerebral infarction, insomnia, acupuncture therapy, poststroke insomnia, cerebrovascular accident, sleeplessness, and so forth. China National Knowledge Infrastructure (CNKI), Wanfang Database, VIP Database, China Biology Medicine (CBM), PubMed, Cochrane Library, Embase, and Web of Science were searched from the establishment of the databases to April 29, 2021, to collect all the randomized controlled trials of acupuncture in the treatment of poststroke insomnia. The search strategy in PubMed is shown in Table S2.

### 2.6. Study Selection and Data Extraction

Two researchers independently screened the literature according to the inclusion and exclusion criteria, excluded the obviously irrelevant ones, and cross-checked the results. In case of any disagreement, the third researcher should participate in the discussion to decide. The following data of the included literature were extracted by Excel 2013: ① clinical research (title, first author, publication date, sample size, gender ratio, and average age), ② intervention measures (treatment regime of the control group, the frequency and course of acupuncture treatment in the experimental group), ③ each risk of bias assessment elements in RCTs, and ④ outcome indicators.

### 2.7. Literature Quality Assessment

Two researchers conducted the evaluation independently according to the criteria of the Cochrane Collaboration tool [15]. If there were disagreements, they would consult with the third researcher. The evaluation contents contained the following: (1) random sequence generation; (2) allocation concealment; (3) blinding of the participants, the treatment plan implementers, and the outcome assessors or personnel; (4) incomplete outcome data; (5) selective reporting of the research results; and (6) other sources of bias.

### 2.8. Statistical Analysis

The Review Manager 5.3 software was used for meta-analysis. Dichotomous variables and continuous variables were analyzed with relative risk (RR) and weighted mean difference (WMD) as efficacy indicators, respectively. The chi-square test and *I*^2^ statistic were used to assess heterogeneity. If *I*^2^ ≤ 50% and *P* ≥ 0.1, it was considered that there was low heterogeneity between the studies. If *P* < 0.1and *I*^2^ > 50%, it was considered that there was great heterogeneity, and we investigated the source of heterogeneity. Because the age, course of disease, and acupoints used in each study were different and those potential clinical heterogeneities could not be completely avoided, we used the random-effects model for meta-analysis. If it was impossible to conduct the combined analysis, descriptive analysis would be used. The results were shown via forest plot and funnel plot was used to evaluate the publication bias of the literature. *P* < 0.05 was statistically significant.

## 3. Results

### 3.1. Results of the Literature Search

**Figure 1:**
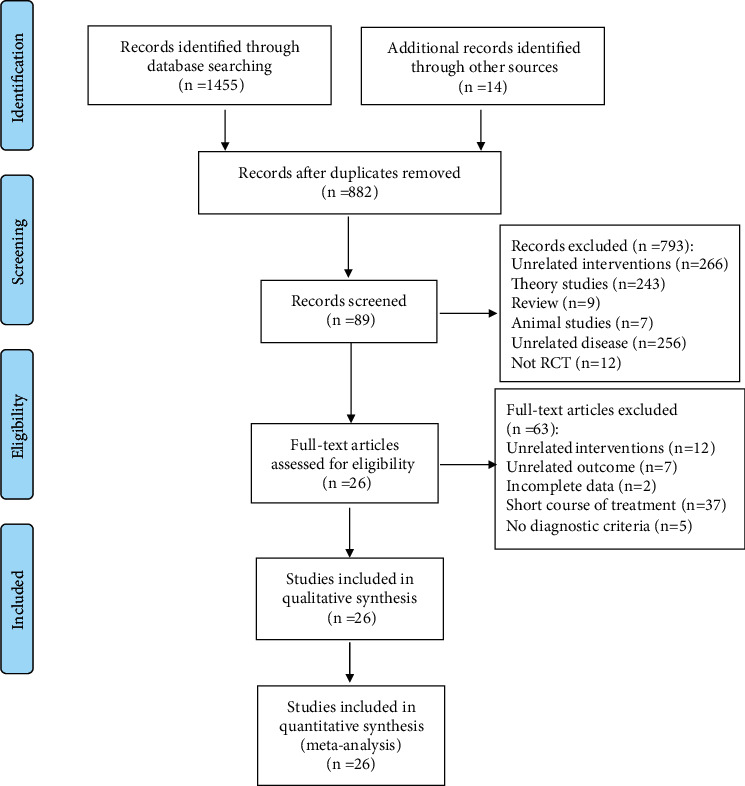
The selection process of the literature.

1455 papers in Chinese and 14 papers in English were identified from the initial retrieval. After screening, a total of 26 RCTs were included. The selection process of the literature is shown in Figure 1.

### 3.2. Basic Characteristics of the Included Studies

A total of 26 RCTs were included, including 1874 cases with 942 in the treatment group and 932 in the control group.  Table 1 displays the characteristics of the included literature. As for the diagnostic criteria of stroke, 15 studies adopted *diagnostic points of various cerebrovascular diseases* [42], 4 studies adopted *guidelines for prevention and treatment of cerebrovascular diseases in China* [43], 3 studies adopted *Chinese guidelines for diagnosis and treatment of acute ischemic stroke* [44], 2 studies adopted *criteria for diagnosis and efficacy assessment of apoplexy* [45], and 2 studies adopted *diagnostic criteria of integrated traditional Chinese and western medicine for cerebral infarction and cerebral hemorrhage* [46]. As for the diagnostic criteria of insomnia, 16 studies adopted *the Chinese Classification of Mental Disorders (CCMD-3)*, *3rd ed.* [47], 6 studies adopted *diagnosis and treatment of insomnia* [48], 2 studies adopted *ICD-10 Classification of Mental and Behavioural Disorders* [49], and 2 studies were based on PSQI > 7.  Table 2 shows the details.

### 3.3. Literature Quality Assessment

**Figure 2:**
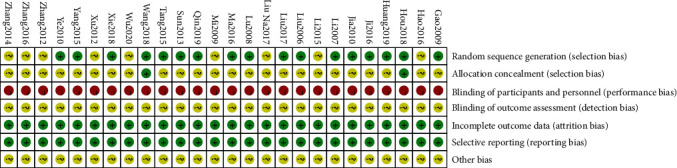
Risk-of-bias assessment results of the included studies.

This study evaluated the literature quality according to the criteria recommended in the Cochrane Handbook for Systematic Reviews of Interventions. 17 studies used random number table, which were assessed as low risk, while 9 studies did not describe the specific grouping methods and were assessed as unclear risk. 24 studies did not describe the specific ways of allocation, which were assessed as unclear risk. 26 studies did not use blind method, so we assessed them as high risk. Besides, all the included RCTs did not describe the blind method in the outcome evaluation and were assessed as unclear risk. All the 26 studies reported the set outcome indicators, which were evaluated as low risk. Meanwhile, all these studies did not describe other biases and were assessed as unclear risk.  Figure 2 shows the quality evaluation of the included literature.

### 3.4. Meta-Analysis Results

#### 3.4.1. Clinical Effective Rate

**Figure 3:**
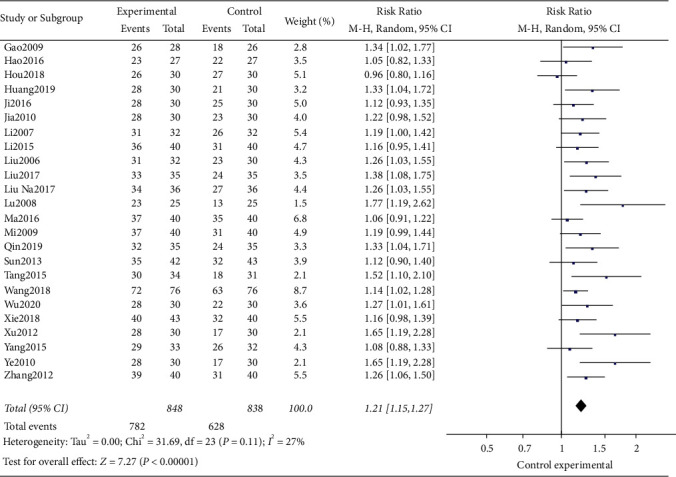
Forest plot of the clinical effective rate between the two groups.

**Figure 4:**
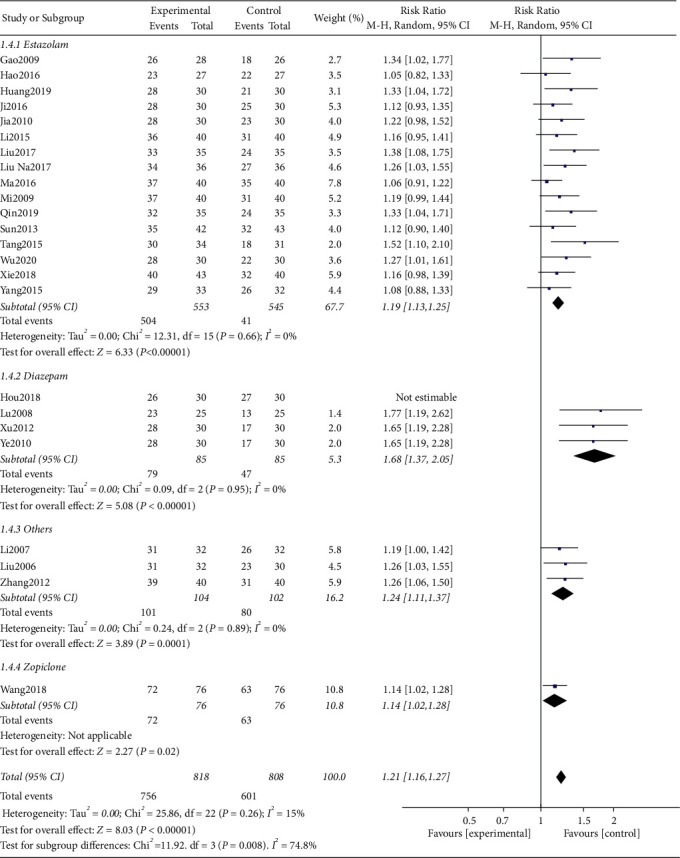
Forest plot of the subgroup analysis in the clinical effective rate between the two groups.

Among the included studies, a total of 24 studies used the clinical effective rate as the evaluation indicator. The test for heterogeneity was not significant (*I*^2^ = 27%, *P*=0.11); the results of meta-analysis showed that the clinical effective rate of acupuncture related therapy was better than that of oral western medicine in the treatment of poststroke insomnia, and the difference was statistically significant ([RR = 1.21; 95% CI: 1.15, 1.27; *P* < 0.00001], Figure 3). In order to further explore the efficacy between acupuncture related therapy and different hypnotics, we subdivided the control group into estazolam group, diazepam group, zopiclone group, and others (the names of hypnotics not mentioned in the studies) according to the different hypnotics in the control group. The test for heterogeneity was significant in diazepam group (*P*=0.0002, *I*^2^ = 85%). When the study of Hou (2018) [18] was excluded, the heterogeneity disappeared (*I*^2^ = 0%, *P*=0.95), suggesting that this study was the source of heterogeneity. After analyzing the original text, it was found that the treatment group added repetitive transcranial acupuncture stimulation (rTAS) on the basis of acupuncture, and there was no statistical significance in the clinical effective rate compared with the control group, which may be the source of heterogeneity. After excluding the source of heterogeneity, a random-effects model was used for meta-analysis of the data. The results showed that the clinical effective rate of the treatment group was better than those of estazolam group [RR = 1.19; 95% CI: 1.13, 1.25; *P* < 0.00001], diazepam group [RR = 1.68; 95% CI: 1.37, 2.05; *P* < 0.00001], others group [RR = 1.24; 95% CI: 1.11, 1.37; *P*=0.0001], and zopiclone group [RR = 1.14; 95% CI: 1.02, 1.28; *P*=0.02] with statistically significant difference (*P* < 0.05, Figure 4).

#### 3.4.2. PSQI Score

**Figure 5:**
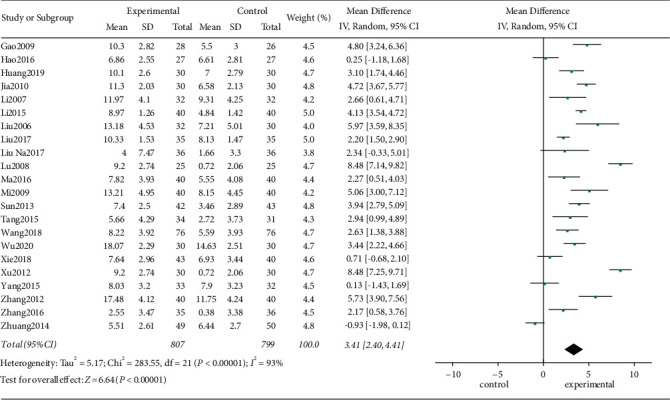
Forest plot of PSQI between the two groups.

**Figure 6:**
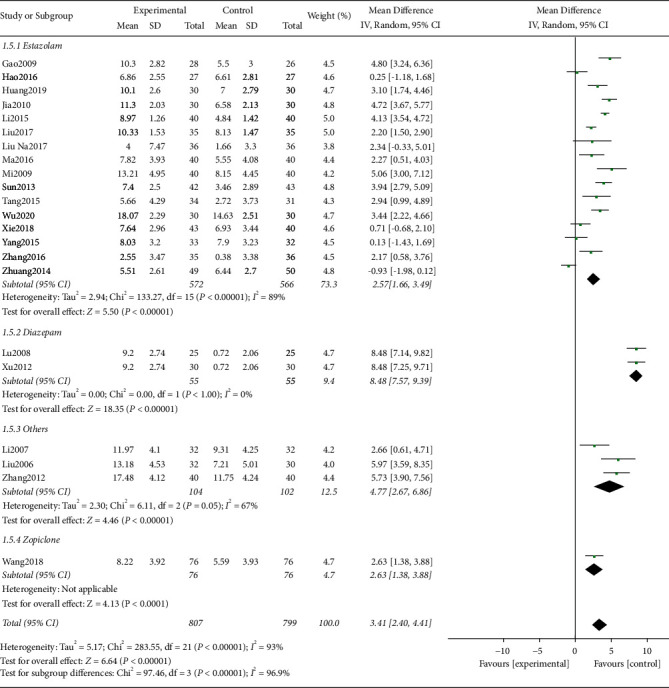
Forest plot of the subgroup analysis in PSQI between the two groups.

Among the included studies, a total of 22 studies used PSQI as the evaluation indicator. The test for heterogeneity was significant between the studies (*I*^2^ = 93%, *P* < 0.00001). We tried to reduce the heterogeneity through subgroup analysis but still could not eliminate the heterogeneity. By analyzing the full text of the included studies, we found that the acupoints and acupuncture methods of each study were not exactly the same, which might be the main reason for heterogeneity. The combined effect size did not change significantly after excluding studies one by one, suggesting that the result was stable. Finally, we used the random-effects model for analysis. The results of meta-analysis showed that there was a significant difference in PSQI between acupuncture related therapy and western medicine in the treatment of poststroke insomnia ([MD = 3.41; 95% CI: 2.40, 4.41; *P* < 0.00001], Figure 5). We also conducted subgroup analysis according to the different hypnotics in the control group, namely, estazolam group, diazepam group, others group, and zopiclone group. The tests for heterogeneity were all significant except in the diazepam group (Figure 6). The random-effects model was used for meta-analysis of the data. The results showed that the PSQI score of the treatment group was better than those of estazolam group [MD = 2.57; 95% CI: 1.66, 3.49; *P* < 0.00001], diazepam group [MD = 8.48; 95% CI: 7.57, 9.39; *P* < 0.00001], others group [MD = 4.77; 95% CI: 2.67, 6.86; *P* < 0.00001], and zopiclone group [MD = 2.63; 95% CI: 1.38, 3.88; *P* < 0.00001] with statistically significant difference (*P* < 0.05, Figure 6).

#### 3.4.3. Adverse Reactions

**Figure 7:**
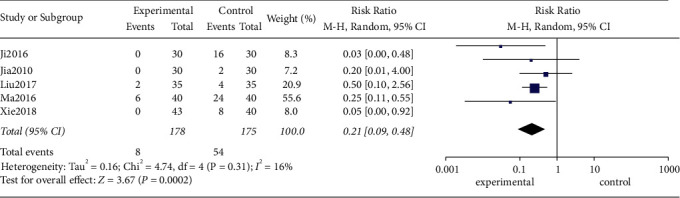
Forest plot of adverse reactions between the two groups.

Five of the included studies reported adverse reactions. The test for heterogeneity showed that there was little heterogeneity between the studies (*I*^2^ = 16%, *P*=0.31), and a random-effects model was used for analysis. The results of meta-analysis revealed that the adverse reactions rate of acupuncture related therapy in the treatment of poststroke insomnia was lower than that of oral western medicine, and the difference was statistically significant ([RR = 0.17; 95% CI: 0.09, 0.48; *P*=0.0002], Figure 7).

### 3.5. Sensitivity Analysis

**Figure 8:**
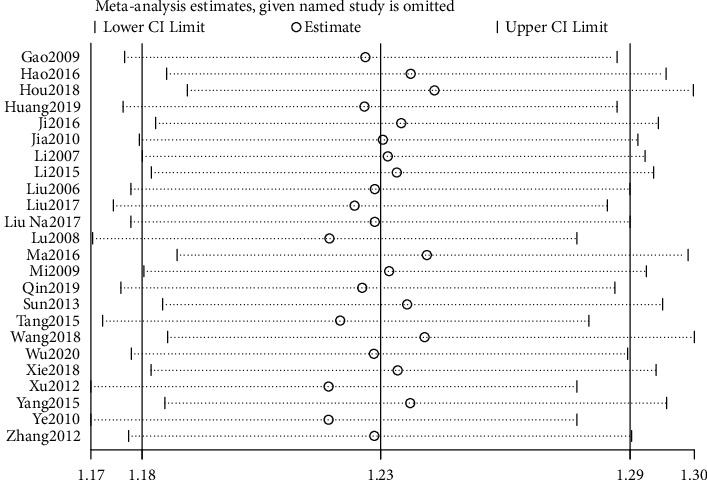
Sensitivity analysis of the total effective rate.

**Figure 9:**
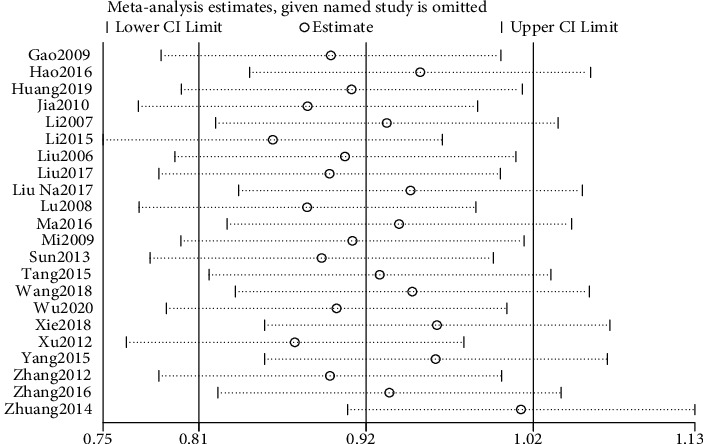
Sensitivity analysis of PSQI.

**Figure 10:**
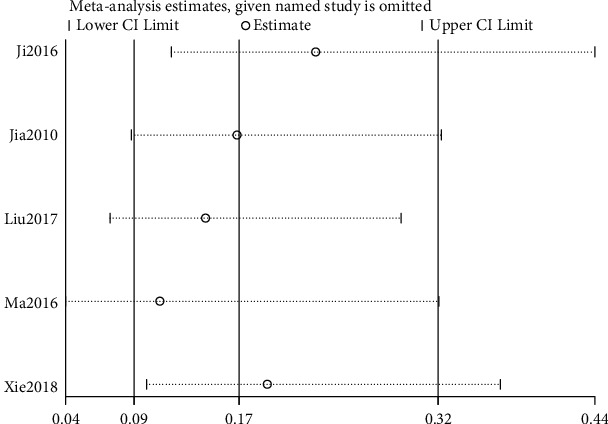
Sensitivity analysis of adverse reactions.

The sensitivity analysis of the above indicators was conducted by one-by-one elimination method to observe the stability of the combined results. After the sensitivity analysis of clinical effective rate, PSQI score, and adverse reactions, it was found that the *P* values of the outcome indicators were less than 0.05 when any literature was excluded, suggesting that the results of these indicators were relatively stable (Figures 8–10).

### 3.6. Publication Bias

**Figure 11:**
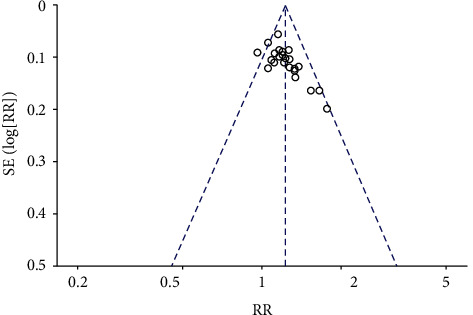
The funnel plot of the effective rate.

In this study, the funnel plot was used to evaluate whether there was potential publication bias in the included literature, and 24 studies recording the effective rate of acupuncture in the treatment of poststroke insomnia were tested. The results showed that although most of the studies were on the upper part of the funnel plot, they scattered asymmetrically, indicating that there might be publication bias (Figure 11).

### 3.7. Overall Quality of Evidence by GRADE

We assessed the available evidence with the GRADE approach. The clinical effective rate was rated as “LOW”; the evidence grade of PSQI score was “LOW”; the evidence grade of adverse reactions was “Very LOW.” We degraded the evidence quality of outcome indicators based on the following reasons: ① Some of the studies did not describe randomization, and none of the studies described blinding of participants and personnel, as well as blinding of outcome assessment. ② Funnel plot test showed publication bias in the results. ③ Downgrading a notch was conducted because the number of the included studies was small, and the confidence interval was wide (Table 3).

## 4. Discussion

Poststroke insomnia is a complication with high incidence in the stroke patients. Long-term sleep disorders will not only reduce the daytime functional activities of the patients but also lower their quality of life obviously and postpone the rehabilitation process of other diseases. Therefore, we should pay full attention to the sleep disorders of stroke patients and treat actively in clinical practice. Previous studies [18, 50] have shown that acupuncture can improve the sleep quality of poststroke insomnia patients. At the same time, it also has the advantages of convenient operation, definite efficacy, and less side effects, which can also be used to treat depression [51], headache [52], diarrhea [53], and other diseases in clinical practice.

In traditional Chinese medicine, the etiology and pathogenesis of insomnia are related to the imbalance of Yin and Yang and the failure of Yang to enter Yin, which is also the etiology and pathogenesis of poststroke insomnia [54, 55]. Modern studies have shown that poststroke insomnia is related to the physiological function of the lesion location [56], neurotransmitters and endocrine peptide hormones [57], social and psychological factors [58], and so forth. Acupuncture has a unique effect on the treatment of poststroke insomnia, which adjusts the function of internal organs and increases the patient's sleep duration and sleep efficiency by stimulating specific acupoints on the patient's body [59, 60]. Modern research has confirmed that acupuncture at some specific acupoints (such as Yintang, Taiyang, and Sishencong) can also promote the increase of some sleep related neurotransmitters in the brain (such as serotonin and *γ*-aminobutyric acid) and reduce the content of sleep inhibitory neurotransmitters (such as norepinephrine) [61]. In addition, warm needle acupuncture, electroacupuncture, and other methods have more obvious intervention on neurotransmitters. The research has shown that, after the intervention of designated acupoints by warm needle acupuncture, there is no significant difference in the content of dopamine (DA) in the rats' hypothalamus, hippocampus, and prefrontal cortex compared with the diazepam group, indicating that warm needle acupuncture has the same effect as the drug in reducing the content of DA [62]. Acupuncture can dredge the meridians, coordinate Yin and Yang, and adjust the internal organs [63]. It can promote the body's Yin and Yang in equilibrium via adjusting the central nervous hormones and transmitters and correcting neurological dysfunction, so as to improve the quality of sleep.

The meta-analysis results of this study found that, in terms of clinical effective rate and PSQI, acupuncture alone or combined with oral medicine had obvious advantages over oral western medicine alone, and the curative effect was better with statistically significant difference between the two groups. In order to further explore the comparison of the curative effect between acupuncture related therapy and different hypnotics, we conducted subgroup analysis according to the different hypnotics in the control group. The results showed that, compared with oral estazolam, diazepam, or zopiclone, acupuncture alone or combined with oral drugs had significant advantages in improving the clinical effective rate and PSQI score. As for safety, a total of 5 articles were reported, and 2 articles of them reported adverse reactions of the patients in the experimental group, but the incidence of adverse reactions was significantly lower than that in the control group. Sensitivity analysis of each of the outcome indicators showed that the results were stable. We evaluated the quality of evidence with the GRADE system; the clinical effective rate was rated as “LOW”; the evidence grade of PSQI score was “LOW”; and the evidence grade of adverse reactions was “Very LOW.”

Limitations of this study are as follows: (1) Most of the included studies are RCTs with small samples, and the literature quality is not high. (2) Due to the different acupoints and their compatibility as well as the needle application time in the experimental group and types and doses of oral drugs in the control group, some results in the study may have clinical heterogeneity. (3) This study only searched the literature in Chinese and English and did not include the ones in other languages, which may have potential publication bias. (4) Since the diagnostic criteria and indicators used in the included studies are inconsistent, it is recommended that future studies adopt the international diagnostic criteria and try to use objective indicators as the outcome indicators to reduce the impact of subjective factors on the results.

In conclusion, acupuncture is indeed effective in the treatment of poststroke insomnia based on the research results. It can improve the PSQI score of the patients and bring high safety to them, which has strong clinical practicability and is suitable for clinical promotion. However, due to the low quality of the literature included in this study, the reliability of providing basis for clinical treatment is still insufficient. It is expected that more large-sample and high-quality clinical RCTs will be carried out to provide more reliable lines of evidence for acupuncture in the treatment of poststroke insomnia.

## Figures and Tables

**Table 1:** Characteristics of the included literature.

Study cohort	Country	No.	Age	Interventions		Course (days)	Outcome
		(C/T)	(C/T)	C	T		
Gao et al., 2009 [16]	China	54 (26/28)	63.40 ± 7.50/64.60 ± 6.20	Estazolam (1 mg, Qd)	Acupuncture (Shuigou-GV26, Shaoshang-LU11, Yinbai-SP1, Daling-PC7, Shenmai-BL62, Fengfu-GV16, Jiache-ST6, Chengjiang-RN24, Quchi-LI11, etc.), Qd	30	①, ②
Hao et al., 2016 [17]	China	54 (27/27)	58.02 ± 7.96/57.71 ± 8.25	Estazolam (0.5–1 mg, Qd)	Acupuncture (Zusanli-ST36, Guanyuan-RN4, Hegu-LI4, Baihui-GV20, Quchi-LI11, etc.)	28	①, ②
Hou et al., 2018 [18]	China	60 (30/30)	62.00 ± 5.00/61.00 ± 5.00	Diazepam (2.5 mg, Qn)	Acupuncture (Baihui-GV20, Ningshen, Neiguan-PC6, Taiyang-EX-HN5, Sanyinjiao-SP6, Zhaohai-KI6, Zusanli-ST36), 40 min, Qd	30	①
Huang et al., 2019 [19]	China	60 (30/30)	59.47 ± 8.34/60.17 ± 10.12	Estazolam (1-2 mg, Qd)	Acupuncture and moxibustion of invigorating spleen and regulating spirit (Zhongwan-RN12, Tianshu-ST25, Zusanli-ST36, etc.) + Yintan-GV29 + Zusanli-ST36 moxibustion, 30 min, 5 times/week, 4 weeks	28	①, ②
Jia, 2010 [20]	China	60 (30/30)	63.10 ± 7.00/62.50 ± 6.40	Estazolam (2 mg, Qd)	Acupuncture (Shenmen-HT7, Sanyinjiao-SP6, Shenting-GV24, Sishencong-EX-HN1, Neiguan-PC6, Taichong-LR3, Taixi-KI3), 20–30 min, 5 times/week	28	①, ②, ③
Ji, 2016 [21]	China	60 (30/30)	65/63	Estazolam (2 mg, Qd)	Acupuncture (Sanyinjiao-SP6, Baihui-GV20, Anmian, Neiting-ST44, Fenglong-ST40, Shenting-GV24, Shenmen-HT7, etc.), 30 min, Qd	28	①, ③
Li, 2007 [22]	China	64 (32/32)	67.30 ± 8.30/69.80 ± 7.10	Diazepam 2.5 mg or estazolam 1 mg, Qd	Acupuncture (Shenmen-HT7, Sanyinjiao-SP6, Shenting-GV24, Sishencong- EX-HN1, Neiguan-PC6, etc) + auricular point: Shenmen, 20∼30 min, 6 times/week	28	①, ②
Li, 2015 [23]	China	80 (40/40)	50.00 ± 3.00/51.10 ± 2.40	Estazolam (2 mg, Qd)	Acupuncture (Shenmai-BL62, Zhaohai-KI6, Dazhui-GV14, Guanyuan-RN4, Taichong-LR3, Quchi-LI11, etc.), 20 min, 5 times/week	28	①, ②
Liu, 2006 [24]	China	62 (30/32)	67.50 ± 8.20/69.90 ± 6.90	Diazepam 2.5 mg or estazolam 1 mg, Qd	Acupuncture (Shenmen-HT7, Sanyinjiao-SP6, Shenting-GV24, Sishencong- EX-HN1, Neiguan-PC6, etc.) + auricular point (Shenmen), 30 min, 5 times/week	28	①, ②
Liu et al., 2017 [25]	China	70 (35/35)	63.02 ± 7.79/62.08 ± 7.82	Estazolam (1 mg∼2 mg, Qd)	Acupuncture (Baihui-GV20, Shenting-GV24, Guanyuan-RN4, Qihai-RN6, Shenmen-HT7, Sanyinjiao-SP6, etc.), 30 min, 5 times/week	28	①, ②, ③
Liu and Zhang, 2017 [26]	China	72 (36/36)	52.08 ± 6.19/52.31 ± 8.26	Estazolam (1 mg, Qd)	Auricular acupuncture (Xin, Shenmen, Neifenmi, Jiaogan, etc.), every 3 days, 2 times/week	28	①, ②
Lu et al., 2008 [27]	China	50 (25/25)	62.40 ± 4.88/61.48 ± 3.72	Diazepam (5 mg, Qd)	Acupuncture (Shenting-GV24, Baihui-GV20, Fengfu-GV16, Shendao-GV11, Shenshu-BL23, Taixi-KI3, Shenmen-HT7, Neiguan-PC6), 30 min, 6 times/week	28	①, ②
Ma et al., 2016 [28]	China	80 (40/40)	63.70 ± 4.94/61.88 ± 5.16	Estazolam (1 mg, Qd)	Acupuncture (Baihui-GV20, Shenting-GV24, Guanyuan-RN4, Qihai-RN6, Shenmen-HT7, Sanyinjiao-SP6, etc.), 30 min, Qd	28	①, ②, ③
Mi et al., 2009 [29]	China	80 (40/40)	63.11 ± 11.96/65.28 ± 10.55	Estazolam (1 mg, Qd)	Acupuncture (Shuigou-GV26, Neiguan-PC6, Sanyinjiao-SP6, JIquan-HT1, Chize-LU5, Weizhong-BL40, Zhongwan-RN12, etc.), 30 min, 5 times/week	28	①, ②
Qin et al., 2019 [30]	China	70 (35/35)	51.80 ± 5.10/52.50 ± 5.30	Estazolam (1 mg, Qd)	Acupuncture (Shendao-GV11, Shuigou-GV26, Shenting-GV24, Shenshu-BL23, Taixi-KI3), 50 min, Qd	28	①, ②
Sun, 2013 [31]	China	85 (43/42)	73.00 ± 10.56/74.83 ± 8.84	Estazolam (1 mg, Qd)	Cowherb seed ear beans (Xin, Shenmen, Pizhixia, etc.), 3 min, Tid)	28	①, ②
Tang and Zhang, 2015 [32]	China	65 (31/34)	59.68 ± 8.73/58.25 ± 9.31	Estazolam (2 mg, Qd)	Acupuncture (Shenting-GV24, Benshen-GB13, Shenmen-HT7, etc.), 30 min, 5 times/week	28	①, ②
Wu et al., 2020 [33]	China	76 (38/38)	59.31 ± 3.51/58.22 ± 3.42	Estazolam (1 mg, Qd)	Estazolam + acupuncture (Baihui-GV20, Shenting-GV24, Yintan-GV29, Sishencong- EX-HN1, etc.), 30 min, Qd, 6 times/week	28	①, ②
Wang et al., 2018 [34]	China	152 (76/76)	64.00 ± 6.00/64.00 ± 6.00	Zopiclone (3 mg, Qd)	Zopiclone + acupuncture (Baihui-GV20, Taiyang-EX-HN5 Zusanli-ST36), 20–30 min, Qd	28	①, ②
Xie, 2018 [35]	China	83 (40/43)	58.15 ± 12.20/56.94 ± 9.83	Estazolam (2 mg, 4–6 times/week)	Acupuncture (Sanyinjiao-SP6, Shenmen-HT7, Sishencong-EX-HN1, Taichong-LR3, Neiguan-PC6, Taixi-KI3, etc), 20–30 min, 6 times/week	28	①, ②, ③
Xu et al., 2012 [36]	China	60 (30/30)	62.40 ± 4.78/62.48 ± 3.66	Diazepam (5 mg, Qd)	Acupuncture (Shenting-GV24, Baihui-GV20, Fengfu-GV16, Shendao-GV11, Sishencong-EX-HN1), 30 min, 6 times/week	28	①, ②
Yang, 2015 [37]	China	65 (32/33)	63.20 ± 7.21/65.20 ± 7.64	Estazolam (2 mg, Qd)	Moxibustion (Dazhui-GV14 to Yaoyangguan-GV3), 45 min, 3 times/week	90	①, ②
Ye et al., 2010 [38]	China	60 (30/30)	62.40 ± 4.88/61.48 ± 3.72	Diazepam (5.0 mg, Qd)	Acupuncture (Shenting-GV24, Baihui-GV20, Fengfu-GV16, Shendao-GV11, Sishencong-EX-HN1), 30 min, 6 times/week	28	①
Zhang, 2012 [39]	China	80 (40/40)	60.4/58.7	Diazepam 2.5 mg or estazolam 1 mg, Qd	Acupuncture (Shenmen-HT7, Baihui-GV20, Shenting-GV24, etc.) + auricular acupuncture (Xin, Pi, Shenmen, Jiaogan), 30 min, 6 times/week	28	①, ②
Zhang and Zhau, 2016 [40]	China	71 (36/35)	74.70 ± 13.10/74.40 ± 9.38	Estazolam	Estazolam + auricular point pressing (Pizhixia, Shenmen, Shenjingshuairuoqu, Zhen, etc.), 3 min, 2 times/week	40	②
Zhuang and Jiang, 2014 [41]	China	99 (50/49)	61.06 ± 8.72/63.88 ± 8.07	Estazolam (1 mg, Qd)	Acupuncture (Sishencong-EX-HN1, Neiguan-PC6, Shenmen-HT7, Laogong-PC8, etc.), 30 min, Qd	30	②

*Note*. “-”: not mentioned; ①: total effective rate; ② Pittsburgh sleep quality index (PSQI); ③ adverse reactions.

**Table 2:** Diagnostic criteria of the included literature.

Study cohort	Country	Stroke	Insomnia
Gao et al., 2009 [16]	China	Diagnostic points of various cerebrovascular diseases	Diagnosis and treatment of insomnia
Hao et al., 2016 [17]	China	Diagnostic criteria of integrated traditional Chinese and western medicine for cerebral infarction and cerebral hemorrhage	The Chinese Classification of Mental Disorders (CCMD-3), 3rd ed.
Hou et al., 2018 [18]	China	Diagnostic points of various cerebrovascular diseases	Diagnosis and treatment of insomnia
Huang et al., 2019 [19]	China	Diagnostic points of various cerebrovascular diseases	The Chinese Classification of Mental Disorders (CCMD-3), 3rd ed.
Jia et al., 2010 [20]	China	Diagnostic criteria of integrated traditional Chinese and western medicine for cerebral infarction and cerebral hemorrhage	The Chinese Classification of Mental Disorders (CCMD-3), 3rd ed.
Ji, 2016 [21]	China	Chinese guidelines for diagnosis and treatment of acute ischemic stroke	ICD-10 Classification of Mental and Behavioural Disorders
Li, 2007 [22]	China	Diagnostic points of various cerebrovascular diseases	Diagnosis and treatment of insomnia
Li, 2015 [23]	China	Diagnostic points of various cerebrovascular diseases	The Chinese Classification of Mental Disorders (CCMD-3), 3rd ed.
Liu et al., 2006 [24]	China	Diagnostic points of various cerebrovascular diseases	The Chinese Classification of Mental Disorders (CCMD-3), 3rd ed.
Liu, 2017 [25]	China	Diagnostic points of various cerebrovascular diseases	The Chinese Classification of Mental Disorders (CCMD-3), 3rd ed.
Liu and Zhang, 2017 [26]	China	Chinese guidelines for diagnosis and treatment of acute ischemic stroke	The Chinese Classification of Mental Disorders (CCMD-3), 3rd ed.
Lu et al., 2008 [27]	China	Criteria for diagnosis and efficacy assessment of apoplexy	The Chinese Classification of Mental Disorders (CCMD-3), 3rd ed.
Ma et al., 2016 [28]	China	Diagnostic points of various cerebrovascular diseases	The Chinese Classification of Mental Disorders (CCMD-3), 3rd ed.
Mi et al., 2009 [29]	China	Diagnostic points of various cerebrovascular diseases	The Chinese Classification of Mental Disorders (CCMD-3), 3rd ed.
Qin et al., 2019 [30]	China	Guidelines for prevention and treatment of cerebrovascular diseases in China	The Chinese Classification of Mental Disorders (CCMD-3), 3rd ed.
Sun, 2013 [31]	China	Diagnostic points of various cerebrovascular diseases	Diagnosis and treatment of insomnia
Tang and Zhang, 2015 [32]	China	Diagnostic points of various cerebrovascular diseases	The Chinese Classification of Mental Disorders (CCMD-3), 3rd ed.
Wu et al., 2020 [33]	China	Guidelines for prevention and treatment of cerebrovascular diseases in China	The Chinese Classification of Mental Disorders (CCMD-3), 3rd ed.
Wang et al., 2018 [34]	China	Chinese guidelines for diagnosis and treatment of acute ischemic stroke	ICD-10 Classification of Mental and Behavioural Disorders
Xie, 2018 [35]	China	Diagnostic points of various cerebrovascular diseases	PSQI > 7
Xu et al., 2012 [36]	China	Criteria for diagnosis and efficacy assessment of apoplexy	The Chinese Classification of Mental Disorders (CCMD-3), 3rd ed.
Yang, 2015 [37]	China	Guidelines for prevention and treatment of cerebrovascular diseases in China	The Chinese Classification of Mental Disorders (CCMD-3), 3rd ed.
Ye et al., 2010 [38]	China	Diagnostic points of various cerebrovascular diseases	Diagnosis and treatment of insomnia
Zhang, 2012 [39]	China	Diagnostic points of various cerebrovascular diseases	Diagnosis and treatment of insomnia
Zhang and Zhou, 2016 [40]	China	Guidelines for prevention and treatment of cerebrovascular diseases in China	The Chinese Classification of Mental Disorders (CCMD-3), 3rd ed.
Zhuang and Jiang, 2014 [41]	China	Diagnostic points of various cerebrovascular diseases	PSQI > 7

**Table 3:** GRADE summary table of the outcome indicators evidence quality.

Acupuncture compared to drug for poststroke insomnia					
Patient or population: patients with poststroke insomnia					
Intervention: acupuncture					
Comparison: drug					
Outcomes	Illustrative comparative risks^*∗*^ (95% CI)		Relative effect (95% CI)	No. of participants (studies)	Quality of the evidence (GRADE)
	Assumed risk	Corresponding risk			
	Drug	Acupuncture			
Clinical effective rate	Study population		RR 1.23 (1.18 to 1.29)	1686 (24 studies)	⊕⊕⊝⊝low^1,2^
	749 per 1000	921 per 1000 (884 to 966)			
	Medium risk population				
	767 per 1000	943 per 1000 (905 to 989)			
PQSI score		The mean PQSI score in the intervention groups was 3.41 higher (2.4 to 4.41 higher)		1606 (22 studies)	⊕⊕⊝⊝low^1,2^
Adverse reactions	Study population		RR 0.17 (0.09 to 0.32)	353 (5 studies)	⊕⊝⊝⊝very low^1,2,3^
	309 per 1000	53 per 1000 (28 to 99)			
	Medium risk population				
	200 per 1000	34 per 1000 (18 to 64)			

^*∗*^The basis for the assumed risk (e.g., the median control group risk across studies) is provided in footnotes. The corresponding risk (and its 95% confidence interval) is based on the assumed risk in the comparison group and the relative effect of the intervention (and its 95% CI). CI: confidence interval; RR: risk ratio; GRADE: working group grades of evidence. High quality: further research is very unlikely to change our confidence in the estimate of effect. Moderate quality: further research is likely to have an important impact on our confidence in the estimate of effect and may change the estimate. Low quality: further research is very likely to have an important impact on our confidence in the estimate of effect and is likely to change the estimate. Very low quality: we are very uncertain about the estimate. ^1^Some of the studies did not describe randomization, and none of the studies described blinding of participants and personnel, as well as blinding of outcome assessment. ^2^Funnel plot test showed publication bias in the results. ^3^Downgrading a notch was conducted because the number of included studies is small, and the confidence interval is wide.

## Data Availability

The data used to support the findings of this study are available from the corresponding author upon request.

## Supplementary Materials

Supplementary Materials Table S1: PRISMA checklist. Table S2: the search strategy in PubMed.
