# Supplementary material for: Efficacy and Safety of Acupuncture in the Treatment of Poststroke Insomnia: A Systematic Review and Meta-Analysis of Twenty-Six Randomized Controlled Trials
Source: Evid Based Complement Alternat Med. 2022 Mar 19;2022:5188311. doi: 10.1155/2022/5188311 (PMC8957466; doi:10.1155/2022/5188311)
Supplement: Supplementary Materials — Table S1: PRISMA checklist. Table S2: the search strategy in PubMed. [file 5188311.f1.zip › 5188311.f1/Table S2 The search strategy in PubMed.docx]

| Number |  |
| --- | --- |
| #1 | Stroke[Mesh] |
| #2 | Cerebrovascular Accident[Title/Abstract]) OR Cerebrovascular Apoplexy[Title/Abstract] OR Apoplexy, Cerebrovascular[Title/Abstract] OR Brain Vascular Accident[Title/Abstract] OR Vascular Accident, Brain[Title/Abstract] OR Apoplexy[Title/Abstract] OR Cerebrovascular Accidents, Acute[Title/Abstract] |
| #3 | #1 OR #2 |
| #4 | Sleep Initiation and Maintenance Disorders"[Mesh] |
| #5 | Disorders of Initiating[Title/Abstract] AND Maintaining Sleep[Title/Abstract] OR DIMS[Title/Abstract] OR Early Awakening[Title/Abstract] OR Insomnia[Title/Abstract] OR Sleep Initiation Dysfunction[Title/Abstract] OR Dysfunction, Sleep Initiation[Title/Abstract] OR Sleeplessness[Title/Abstract] |
| #6 | #4 OR #5 |
| #7 | #3 AND #6 |
| #8 | Post-stroke Insomnia[Title/Abstract] OR sleep disordered after stroke[Title/Abstract] OR Post-stroke Sleep disorsers[Title/Abstract] |
| #9 | #7 OR #8 |
| #10 | Acupuncture[Mesh] |
| #11 | Pharmacopuncture[Title/Abstract] OR (Acupotomy[Title/Abstract] OR Acupotomie[Title/Abstract] OR Acupoint[Title/Abstract] OR Acusector[Title/Abstract] OR Electroacupuncture[Title/Abstract] OR electro-acupuncture[Title/Abstract] OR electrical acupuncture[Title/Abstract] OR Ear acupuncture[Title/Abstract] OR needle[Title/Abstract] OR needling[Title/Abstract] OR acupoint injection[Title/Abstract] OR puncturing collateral[Title/Abstract] OR bloodletting[Title/Abstract] OR bleeding therapy[Title/Abstract] OR quick puncture[Title/Abstract] |
| #12 | #10 OR #11 |
| #13 | #9 AND #12 |
